# Complications from radiotherapy for breast cancer

**DOI:** 10.1590/S1516-31802011000200012

**Published:** 2011-03-03

**Authors:** Gustavo Nader Marta, Samir Abdallah Hanna, Eduardo Martella, João Luis Fernandes da Silva

**Affiliations:** I MD. Resident in Radiotherapy, Oncology Center, Hospital Sírio-Libanês, São Paulo, Brazil.; II MD. Radiation therapy specialist and attending physician, Department of Radiation Oncology, and preceptor of radiotherapy residency, Hospital Sírio-Libanês, São Paulo, Brazil.; III MD, PhD. Radiation therapy specialist and attending physician, Department of Radiation Oncology, Hospital Sírio-Libanês, São Paulo, Brazil.; IV MD. Radiation therapy specialist and coordinator, Department of Radiation Oncology, Hospital Sírio-Libanês, São Paulo, Brazil.

Dear editor,

A search through the medical literature discloses relatively little information with a high level of evidence regarding the complications from radiotherapy (RT) for breast cancer. In a single study published in 2001,^1^ an attempt was made to group all published papers on acute toxicity following breast RT, but the techniques and radiation doses in the studies analyzed by these authors had not been prescribed uniformly. Nevertheless, historically, most published papers used conventional RT and did not use any currently accepted quality control for the treatments prescribed.^1^

Technological advances in RT have practically eliminated radiodermatitis as a common complication in settings in which the skin does not need to be irradiated, such as treatments for prostate or lung cancer. On the other hand, breast RT still requires caution in this regard, because the skin must be deliberately irradiated. There are lymph vessels communicating between the skin and the breast parenchyma, thus making the skin a target area for treatment. Invariably, therefore, skin symptoms will be present to a certain extent during RT delivery.

The incorporation of modern RT into treatments for breast cancer has enabled significant gains in this respect, and the literature shows that there has been a drastic reduction in the incidence of radiodermatitis through the use of this technology, as demonstrated in several randomized studies. ^2^–^4^ During RT delivery, however, most patients with breast cancer experience acute adverse reactions that are usually not disabling enough to affect their daily activities. Acute skin reactions are most frequently observed in this setting, and itching, skin dryness and erythema with or without desquamation are the most common symptoms. When scaling occurs, it is preferentially located in natural skinfolds, such as the inframammary areas and the axillae. In 10% to 30% of cases, radiodermatitis leads to temporary interruption of treatment delivery, ^5^ which negatively affects our understanding of radiobiology. The main factors correlated with the intensity of radiodermatitis are dose, energy, dose fractionation and technique, breast volume, use of concurrent chemotherapy, appropriateness of self-care and nutritional status of the patient. ^5^

The Radiation Therapy Oncology Group (RTOG) scale^6^ grades the effects of RT on the skin using the following system:

- Grade 0 - No reaction
- Grade 1 - Weak erythema, painless epilation, dry desquamation, decreased sweating and pruritus
- Grade 2 - Moderate erythema, bright exudative dermatitis in plaques and moderate edema
- Grade 3 - Exudative dermatitis beyond the skinfolds and intense edema
- Grade 4 - Ulceration, hemorrhage and necrosis

Although there are no consensus guidelines to be followed during the course of RT, some simple measures are recommended for patients treated at the Department of Radiation Oncology of Hospital Sírio-Libanês:

- Clean up the irradiated skin with cold/tepid water and ordinary soap;
- Keep your nails clean and short and avoid rubbing or scratching the irradiated site;
- Take special care when shaving/waxing or performing any other type of potentially aggressive maneuver to the skin under treatment;
- Do not apply chemicals, perfumes, ice packs, hot compresses or any other material on the irradiated skin without firstly consulting the RT staff;
- Do not expose the irradiated area to the sun;
- Use a comfortable, preferably seamless bra, and do not overtighten it across the skin;
- Preferably use cotton clothing;
- Moisturize the skin after each RT session, using a cream recommended by the RT staff;
- Apply cold compresses with chamomile tea on irritated irradiated skin.

After the end of RT, although the acute radiodermatitis tends to regress within 10 to 15 days, the irradiated region remains hyperemic for six to nine months due to vasodilatation, and only gradually returns towards normal after this time. However, there may be esthetic changes resulting from breast volume loss, fibrosis and retraction. Although widely used in most centers, the effectiveness of topical therapies for minimizing the duration and severity of skin reactions has not been proven.^7^ A randomized study conducted by the RTOG showed no significant differences in the incidence of radiodermatitis when a topical formulation (Biafine, a combination of alginic, sorbic and stearic acids) was compared with general supportive care, ^8^, ^9^ despite preliminary evidence suggesting that the use of this formulation could avoid treatment interruptions due to acute toxicity.^6^ The use of compounds containing *Calendula officinalis* has also been assessed, and a randomized study has shown their benefit in comparison with the application of trolamine salicylate.^7^, ^10^ However, the benefit of these compounds has not been proven in placebo-controlled studies.

Other expected acute reactions from RT on the breast are fatigue, pain and localized edema.^5^ When the supraclavicular fossa is included in the radiation field, attention should be paid to any appearance of dysphagia, despite the fact that modern RT planning makes it possible to treat the area of interest with very little irradiation of other organs.^5^

Among the chronic side effects from RT on the breast, the most frequent is actinic pneumonitis, which typically stabilizes one year after the treatment.^8^ Within this context, radiographic changes are more common than clinical manifestations. Some studies have suggested that concurrent use of chemotherapy increases the risk of pneumonitis, as demonstrated by the Joint Center for Radiation Therapy, of Harvard Medical School (1% for RT versus 8% for RT plus chemotherapy).^8, 11^

Studies on conventional RT applied to patients with breast cancer have shown that inclusion of the heart in the field of treatment increases the long-term risk of cardiovascular death due to acute myocardial infarction.^12^ However, the results from studies using modern RT techniques have not shown any increased risk of cardiac morbidity and mortality after 10 to 20 years of follow-up.^13, 14^

Nowadays, lymphedema is no longer seen as a complication of RT, since the axilla is no longer included in the RT field. Brachial plexopathy and rib fracture have been reported but are extremely rare events.^15^ Finally, again due to enhanced RT techniques and equipment, the risk of a second malignancy is minimized by reducing the volume of healthy tissues within the treatment field. The overall risk of radiation-induced cancer is approximately 0.2% every 10 years.^1,16^

With the rapid advances of medical specialties that have been seen recently, it is imperative to demystify the effects of RT and to widely publicize and recognize the need for good quality care. Participation by radiation oncologists as active members of the multidisciplinary team, in order to define the therapeutic strategy from its outset, is essential for ensuring these goals.
